# Impact of Attached File Formats on the Performance of ChatGPT-4 on the Japanese National Nursing Examination: Evaluation Study

**DOI:** 10.2196/67197

**Published:** 2025-01-22

**Authors:** Kazuya Taira, Takahiro Itaya, Shuntaro Yada, Kirara Hiyama, Ayame Hanada

**Affiliations:** 1 Human Health Sciences, Graduate School of Medicine, Kyoto University, 53, Shogoinkawara-cho, Sakyo-ku, Kyoto, 606-8507, Japan, 81 0757513927; 2 Department of Healthcare Epidemiology, Graduate School of Medicine and Public Health, Kyoto University, Kyoto, Japan; 3 Graduate School of Science and Technology, Nara Institute of Science and Technology, Ikoma, Japan; 4 Faculty of Library, Information and Media Science, University of Tsukuba, Tsukuba, Japan

**Keywords:** nursing examination, machine learning, ML, artificial intelligence, AI, large language models, ChatGPT, generative AI

## Abstract

This research letter discusses the impact of different file formats on ChatGPT-4’s performance on the Japanese National Nursing Examination, highlighting the need for standardized reporting protocols to enhance the integration of artificial intelligence in nursing education and practice.

## Introduction

Numerous generative artificial intelligences (AIs), exemplified by all versions of ChatGPT [1] and Llama [2], have been developed using large language models and evaluated in health care, particularly in nursing education [3,4], successfully passing national nursing examinations in several countries [5,6]. Generative AIs are evolving to handle multimodal information, including text and images [1]. However, previous evaluations have not assessed the impact of file formats [5,6].

Prompts, particularly long ones, can affect response accuracy owing to potential context loss or exceeded token limits [7-9]. In this study, we hypothesized that the file format attached to prompts could affect the results of nursing research that uses generative AI and aimed to evaluate its impact on ChatGPT-4’s performance on the Japanese National Nursing Examination. The findings of this study would be useful for improving the quality of reports on future nursing research that uses generative AI.

## Methods

### Ethics Approval

This study did not require ethical approval or informed consent, as the data analyzed were obtained from a published database from the Ministry of Health, Labour and Welfare.

### Generative AI Model

We used the original, unmodified GPT-4 (gpt-4‐1106-preview, accessed March 2024) without additional training, tuning, or data. ChatGPT, launched by OpenAI in 2022, with GPT-4 released in March 2023, is currently widely used.

### Input Data

The dataset included all 50 basic knowledge questions from the 2023 Japanese National Nursing Examination, along with 190 general questions. The passing standard for these basic knowledge questions was approximately 80%. ChatGPT-3.5 has consistently failed to meet this standard [4], leading us to consider whether performance might vary based on file format. Questions were prepared in TEXT (.txt), DOCX (.docx), PDF (.pdf), and IMAGE (.jpg) formats and in a format that directly described all questions in the prompt (PROMPT-ONLY format). Although other formats, including CSV, JSON, XML, and Markdown, could be used to present questions and choices, we excluded them to maintain consistency and focus on more common formats.

### Prompt Engineering

The prompts for each file format are summarized in Textbox 1.

Textbox 1. **Prompts provided to ChatGPT-4. The files (mentioned at the end of the prompt for TXT, DOCX, PDF, and JPG formats) were made viewable via OpenAI’s application programming interface (API) function: ASSISTANT (type = retrieval).**

**<Prompt for PROMPT-ONLY format>**

*You are an expert in the field of nursing. Answer the given questions briefly and numerically. {Question number}. {Question}. Options: (1) {Option 1}, (2) {Option 2}, (3) {Option 3}, (4) {Option 4}*

**Example:** 1. Which vessel sends blood from the fetus to the placenta in the fetal circulation? Options: (1) Common carotid artery, (2) Pulmonary artery, (3) Umbilical artery, and (4) Umbilical vein.

**<Prompt for TXT, DOCX, PDF, and JPG formats>**

*You are an expert in the field of nursing. Answer briefly and numerically all questions given by the file.*

### Data Analyses

Prompts for all formats were processed for 100 iterations each; the median and IQR of the percentage of correct answers were calculated. Differences among the percentages of correct answers by the attached file format were compared using the Kruskal-Wallis test and Dann-Bonferroni test. Statistical analyses were performed using Python (version 3.11.4) with the *pandas* (version 1.5.3) and *matplotlib* (version 3.7.1) libraries.

## Results

**Figure 1.**
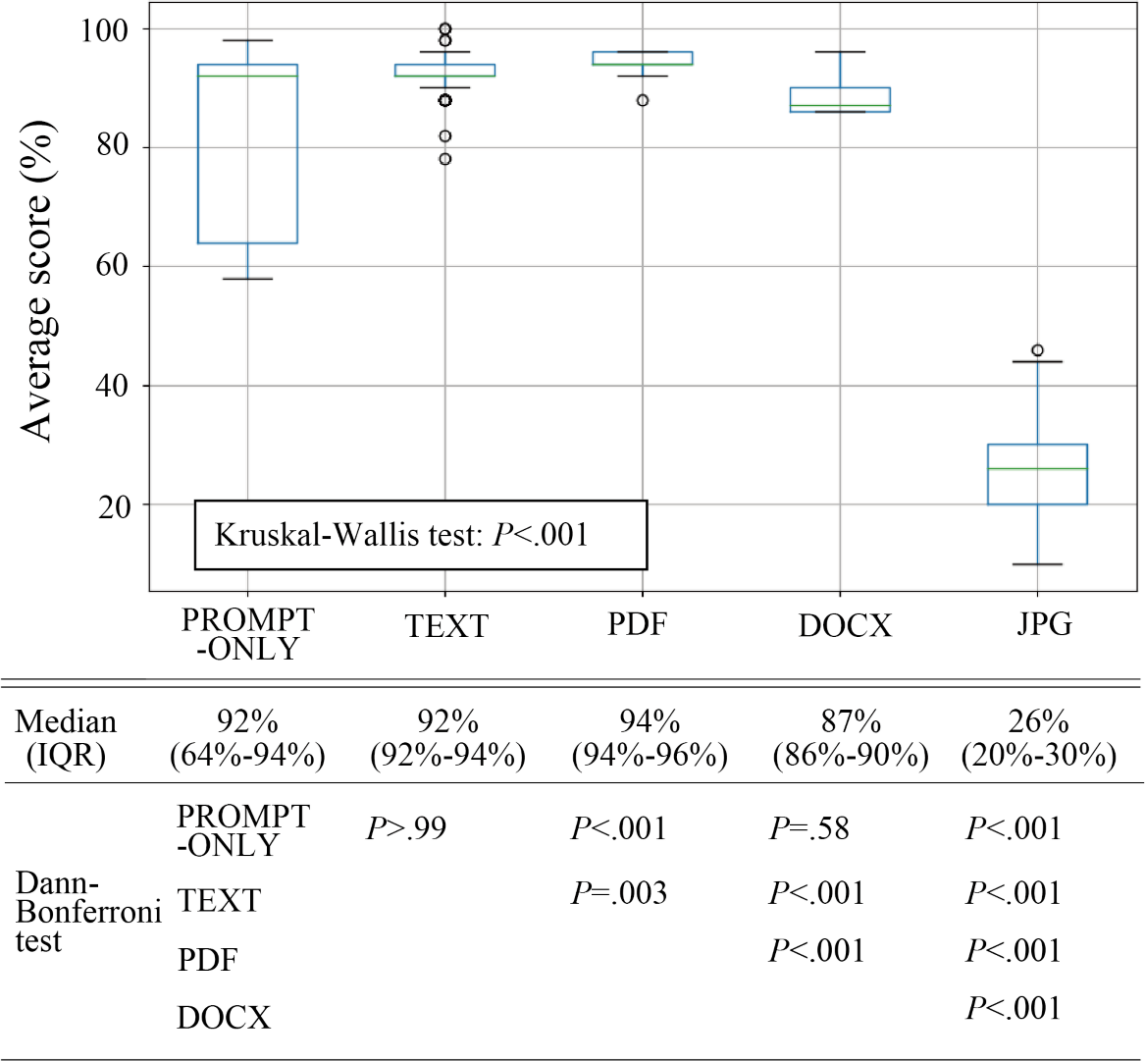
Performance evaluation of ChatGPT-4 on the Japanese National Nursing Examination by the attached file format. Outliers, shown as dots, are values below Q1 – 1.5 or above Q3 + 1.5 in the IQR.

The median percentages of correct answers were 92% (IQR 64%‐94%), 92% (IQR 92%‐94%), 94% (IQR 94%‐96%), 87% (IQR 86%‐90%), and 26% (IQR 20%‐30%) for PROMPT-ONLY, TEXT, PDF, DOCX, and JPG formats, respectively. The differences between the attached formats were statistically significant in all pairs (*P*<.01) except for the PROMPT-ONLY versus TEXT and PROMPT-ONLY versus DOCX pairs (Figure 1).

## Discussion

ChatGPT-4’s performance on the Japanese National Nursing Examination varied significantly across file formats. The best performance was observed with PROMPT-ONLY, TEXT, and PDF formats (median scores >92%), followed by DOCX (87%), and the worst performance was with JPG (26%). The PROMPT-ONLY format exhibited a larger IQR and more variability than TEXT, PDF, and DOCX formats. JPG’s poor performance highlights a significant limitation of generative AI, which excels at processing digital text but struggles with interpreting text from images. This “visual comprehension” gap has critical implications for AI applications involving nondigital text sources. The variability in PROMPT-ONLY performance may reflect reduced accuracy with longer prompts [7,8].

Therefore, to prepare for a future where generative AI is integrated into nursing practice and education [10], it is crucial to understand the interaction between humans and generative AI, including the impact of input file formats. Additionally, it is essential to report the following aspects in a standardized manner:

- Name and version of the generative AI model
- Presence of additional training, tuning, or knowledge transfer
- Prompt design and attached file formats
- Response generation parameters, including the number of iterations, temperature settings, and maximum token count
- Execution environment (if applicable)

However, as we only examined ChatGPT-4’s performance on the Japanese National Nursing Examination and the impact of major file formats, investigations on other formats and AI models are warranted. Particularly, evaluating the performance of AI that specializes in image processing and image formats other than JPG and expanding the evaluations to include national nursing examinations in other countries and clinical questions in practice will be important in future research.

## Abbreviations

AI: artificial intelligence
